# Comparative Metabolome and Transcriptome Analyses Reveal Molecular Mechanisms Involved in the Responses of Two *Carex rigescens* Varieties to Salt Stress

**DOI:** 10.3390/plants13212984

**Published:** 2024-10-25

**Authors:** Yiming Wu, Kai Zhu, Chu Wang, Yue Li, Mingna Li, Yan Sun

**Affiliations:** 1 Institute of Animal Sciences, Chinese Academy of Agricultural Sciences, Beijing 100193, China; 2 College of Grassland Science and Technology, China Agricultural University, Beijing 100193, China

**Keywords:** *Carex rigescens*, salt stress, metabolome profiling, transcriptome analysis, amino acid biosynthesis, phenylalanine biosynthesis and metabolism, phenylpropanoid biosynthesis

## Abstract

Salt stress severely inhibits crop growth and production. The native turfgrass species *Carex rigescens* in northern China, exhibits extraordinary tolerance to multiple abiotic stresses. However, little is known about its specific metabolites and pathways under salt stress. To explore the molecular metabolic mechanisms under salt stress, we conducted metabolome analysis combined with transcriptome analysis of two varieties of *Carex rigescens* with differing salt tolerances: salt-sensitive Lvping NO.1 and salt-tolerant Lvping NO.2. After 5 days of salt treatment, 114 and 131 differentially abundant metabolites (DAMs) were found in Lvping NO.1 and Lvping NO.2, respectively. Among them, six amino acids involved in the amino acid biosynthesis pathway, namely, valine, phenylalanine, isoleucine, tryptophan, threonine, and serine, were accumulated after treatment. Furthermore, most DAMs related to phenylalanine biosynthesis, metabolism, and phenylpropanoid biosynthesis increased under salt stress in both varieties. The expression profiles of metabolism-associated genes were consistent with the metabolic profiles. However, genes including *HCT*, *β-glucosidases*, and *F5H*, and metabolite 4-hydroxycinnamic acid, of the two varieties may account for the differences in salt tolerance. Our study provides new insights into the mechanisms underlying salt tolerance in *Carex rigescens* and reveals potential metabolites and genes to improve crop resilience to saline environments.

## 1. Introduction

Soil salinity is a critical abiotic stress and a major barrier to plant growth [1]. Approximately 20% of all cultivated lands and 33% of irrigated agricultural areas globally are impacted by salinity [2,3]. The problem is particularly severe in China [4], which ranks third in the world in terms of land area affected by soil salinization (approximately 37 million hectares) [5].

Salt stress triggers three classes of stress: osmotic, ionic, and secondary [6]. Several metabolites have been shown to mitigate these stresses. For instance, branched-chain amino acids, namely, leucine, isoleucine, and valine, may enhance plant salt tolerance by scavenging reactive oxygen species [7], and ferulic acid might help to enhance salt tolerance via its strong antioxidant properties [8]. Hence, exploring the crucial metabolites and the molecular mechanisms by which they confer salt stress tolerance is of great importance for developing salt-tolerant plant and crop varieties [9].

As a vital vegetative ground cover globally, turfgrass not only serves ornamental and recreational purposes [10]; some turfgrass species exhibit great tolerance and adaptability to saline environments [11,12]. To investigate the molecular mechanisms and explore changes in genes expression under abiotic stress, high-throughput sequencing technology was used to obtain the gene expression of several crops including rice (*Oryza sativa*) [13], wheat (*Triticum aestivum* L.) [14], and millet (*Panicum miliaceum* L.) [15]. Recent studies have applied metabolomics combined with chromatography-mass spectrometry (GC-MS) [16] to explore the salt tolerance of turfgrass species. For instance, Hu et al. found that accumulation of metabolites involved in carbon and nitrogen metabolism and the tricarboxylic acid cycle in the sink roots of bermudagrass (*Cynodon dactylon*) under salinity might contribute to salt tolerance using GC-MS approaches [17]. Following metabolism analysis, Ye et al. suggested that bermudagrass implemented different measures to cope with different pH and ionic values [18]. Li et al. revealed that sugar and amino acid accumulation in response to γ-Aminobutyric acid application might be responsible for salt tolerance in creeping bentgrass cv. Penncross (*Agrostis stolonifera*) [19]. In salt-tolerant seashore paspalum (*Paspalum vaginatum* Sw.) roots, enhancement of lipid metabolism amino acid metabolism may be responsible for salt tolerance [20]. These studies partially elucidated the salt tolerance mechanisms of several turfgrass species and identified specific metabolites involved in the response to salinity stress.

Furthermore, as other various biotic or abiotic stress circumstances, changes in metabolites in response to stress correspond with changes in gene transcription. Numerous studies have demonstrated that genes involved in the phenylpropanoid biosynthesis and amino acid metabolism pathways are induced under salt stress including those encoding *phenylalanine ammonia-lyase* (*PAL*), *chalcone synthase*, *caffeic acid 3-O-methyltransferase* (*COMT*), *threonine dehydratase*, and *branched-chain amino acid aminotransferase* [21,22,23]. However, clarifying the detailed mechanisms of salt-induced responses remains challenging.

*Carex rigescens* (Franch.) V. Krecz. (*C. rigescens*), a native cool-season perennial turfgrass, is widely distributed in northern China [24]. Because of its excellent ability to adapt to multiple abiotic stresses, especially salt stress, its property of low maintenance and its potential value as a lawn species in barren or arid soil, and its ecological absorption and restoration, *C. rigescens* has been highly valued in afforestation and improvement efforts in both urban and country areas affected by salinization [25,26]. *C. rigescens* Lvping NO.2, an original germplasm resource from a seashore in Huanghua, Hebei province, China, was assessed to be tolerant to a high salt stress of a 300 mmol/L NaCl concentration treatment, whereas Lvping NO.1, as an original germplasm resource from Beijing, China, was used as a comparative salt-sensitive material [27,28]. In recent years, our group has studied different aspects of the response to salt stress and underlying tolerance mechanisms in *C. rigescens*, including responses at the growth and physiological [27,28], transcriptomic [24,27], and lipidomic [29] levels. Candidate secondary metabolism pathway genes revealed by these studies in Lvping NO.1, such as *CrCOMT* [26] and *CrUGT87A1* [30], have been verified to be important for salt tolerance regulation, demonstrating the importance of the secondary metabolism pathway in responding to salt stress. Excavating salt-induced genes and metabolites could improve the development of turfgrass and crop cultivar breeding, which could directly or indirectly enhance the ecological management. However, although we have made initial progress in exploring the salt stress mechanisms in *C. rigescens*, how the metabolome analysis combines gene regulations is still largely unknown under salt treatment.

In this study, our objective was to investigate the vital root metabolites, pathways, and genes, and their potential molecular mechanisms associated with salt stress tolerance in *C. rigescens*. CG-MS approaches and high-throughput sequencing analyses were performed using two *C. rigescens* varieties with different salt sensitivities: Lvping NO.1, which is salt-sensitive, and Lvping NO.2, which is salt-tolerant. CG-MS analysis revealed 114 and 131 differentially abundant metabolites (DAMs) in Lvping NO.1 and Lvping NO.2, respectively. Our findings can help to identify crucial metabolites and the corresponding genes involved in salt stress response and help to clarify the salt tolerance mechanisms in *C. rigescens* and the crop resilience to saline environments.

## 2. Materials and Methods

### 2.1. Plant Materials and Salt Treatment

Two varieties of *C. rigescens*, salt-sensitive Lvping NO.1 and salt-tolerant Lvping NO.2, used in this study were stored and provided by the College of Grassland Science and Technology, China Agricultural University. Previous studies [27,28] confirmed their salt sensitivity traits. Seeds were pre-treated with 20% NaOH [31] for 40 min, then rinsed to neutral pH. They were germinated in containers (17.5 × 12.5 × 13.5 cm) filled with equal parts nutrient soil and vermiculite, and grown in a greenhouse (16 h at 25 °C/8 h at 20 °C day/night cycle, with a light intensity of 7000 lux).

After 35 days, healthy seedlings were transferred to half-strength Hoagland’s solution for five days. At 40 days, seedlings were divided into two groups. The treatment group was transferred to another container (20 cm × 13 cm × 6.5 cm, length × width × height) containing half-strength Hoagland’s solution and gradually exposed to increasing NaCl concentrations (50 mM increments daily) until reaching 300 mM, then cultivated for five more days. The control group remained in half-strength Hoagland’s solution. Post-treatment, roots were rinsed and dried. For transcriptomic analysis, three replicates per variety/treatment were used; for metabolomic analysis, six replicates were used.

### 2.2. Nontargeted Metabolic Profiling Analysis

Metabolome profiling and data analysis were performed at Majorbio (Majorbio, Shanghai, China) following their standard procedures. The root samples (60 mg), 40 μL of an internal standard (L-2-chloro-phenylalanine, 0.3 mg/mL), 360 μL pre-chilled methanol, and two small steel balls were placed in a tube. Then the samples were ground at 60 Hz for 2 min and ultrasonic extraction was performed for 30 min. Next, 200 μL of chloroform was added to the samples, which were swirled at 60 Hz for 2 min. After 400 μL distilled water was added, the sample was swirled at 60 Hz for 2 min, and ultrasonic extraction was performed for 30 min. Finally, after low temperature centrifugation (13,000 rpm, 4 °C) for 15 min, 200 μL of supernatant was placed in a glass derivative bottle, and a centrifugal concentration dryer was used to dry the samples. After derivatization, the samples were injected into a mass spectrometer using splitless injection for GC–MS analysis.

GC–MS analysis [32] was performed with an Agilent 7890A-5975C system (Agilent, Santa Clara, CA, USA), with sample separation achieved on an HP-5MS Capillary Column (30 m × 0.25 mm × 0.25 μm, Agilent J&W Scientific, Folsom, CA, USA). Helium was used as the carrier gas at 1 mL/min. MS conditions included an inlet temperature of 260 °C, an electron bombardment ion source with a temperature of 230 °C, quadrupole temperature of 150 °C, and electron energy of 70 eV, and scanning from *m*/*z* 50–600. Samples were analyzed randomly to mitigate instrument signal fluctuations, and quality control samples were analyzed after every ten samples. GC-MS data were processed using ChromaTOF (version 4.34, LECO, St. Joseph, MI, USA) software, where metabolites were identified via the NIST and Fiehn databases, and a three-dimensional data matrix was generated in CSV format.

The normalized data matrix was analyzed using SIMCA-P+14.0 software (Umetrics, Umeå, Sweden). Principal component analysis (PCA) was first used to assess sample distribution and analysis stability, followed by supervised OPLS-DA to evaluate metabolic profile differences. Variables with a VIP score > 1 were considered significant, and model quality was validated using seven rounds of interactive validation and 200 response ranking tests. Differentially abundant metabolites (DAMs) were identified using multidimensional OPLS-DA and single-dimensional analysis (Student’s *t*-test) with criteria of VIP > 1, *p* < 0.05, and log2 FC > 1.2. Annotated DAMs were further analyzed using MetaboAnalyst (https://www.metaboanalyst.ca/ (accessed on 10 May 2018)), and the ID conversion function of the Kyoto Encyclopedia of Genes and Genomes (KEGG) compound website (https://www.kegg.jp/kegg/pathway.html (accessed on 10 May 2018)).

### 2.3. RNA Extraction and Sequencing

Total RNA was extracted from root samples using an Eastep™ Total RNA Extraction Kit (Promega, Beijing, China). RNA quality was verified via 1% RNase-free agarose gel, Nanodrop (Implen, Munich, Germany) and Agilent 2100 Bioanalyzer (Agilent Technologies, CA, USA). High-quality mRNA was used to synthesize cDNA. Double-stranded cDNA was purified, end-repaired, A-tailed, and ligated to sequencing adapters. After size selection and PCR amplification, the library was quantified using a Qubit 2.0 and validated with an Agilent 2100. Quantitative Real-Time PCR (qPCR) accurately quantified the effective concentration of the library to ensure that the quality was sufficient (effective concentration > 2 nM). Libraries were pooled according to the effective concentration and target data volume requirements for Illumina HiSeq sequencing. Raw reads were processed to clean data by removing adapters, reads with high proportions of N bases (>10%), and low-quality reads (Qphred ≤ 20 for >50% of the total).

Clean data were assembled using Trinity software (v2.4.0) [33], and transcript ex-pression patterns were clustered using Corset (v1.05) [34] software (Table S2). RSEM software (RNA-Seq by Expectation Maximization, v1.2.15) [35] with bowtie2 was used to align clean reads to the reference sequence, and gene expression levels were calculated as Fragments Per Kilobase of transcript sequence per Million mapped reads (FPKM) [36]. Differentially expressed genes (DEGs) were identified using DESeq (1.10.1) [37] with an adjusted *p* < 0.05. KEGG analysis (http://www.genome.jp/kegg/ (accessed on 22 July 2022)) was conducted using KOBAS [38] software (KO-Based Annotation System, v2.0.12). Raw sequence reads were uploaded to a public database (https://www.ncbi.nlm.nih.gov/bioproject/?term=PRJNA860334 (accessed on 22 July 2022)).

### 2.4. Quantitative Reverse Transcription PCR

To validate the RNA-seq data, nine and eight genes in Lvping NO.1 and Lvping NO.2 were randomly selected for quantitative reverse transcription PCR (qRT-PCR) assays. Every gene was randomly chosen from amino acid biosynthesis, phenylalanine biosynthesis, metabolism, and phenylpropanoid biosynthesis pathways. Primers for these genes and the internal reference gene *CreIF-4α* [24], which were designed using Primer Premier 6 software, are shown in Table S3.

cDNA synthesis was carried out using HiScript III All-in-one RT SuperMix Perfect for qPCR (Vazyme, Nanjing, China) with high-quality RNA. Gene expression analysis was then performed according to the manufacturer’s instructions for Taq Pro Universal SYBR qPCR Master Mix (Vazyme, China) on an ABI 7300 qPCR System (Applied Biosystems, Waltham, CA, USA). The qPCR program consisted of initial denaturation at 95 °C for 2 min, followed by 40 cycles of 95 °C for 5 s and 60 °C for 30 s, 95 °C for 5 s, 65 °C for 5 s, and 95 °C for 5 s. Gene expression levels were calculated using the 2^−ΔΔCt^ method [39]. Statistical analysis to determine significant differences was performed using GraphPad Prism 9 software.

## 3. Results

### 3.1. Analysis of DAMs in Two C. rigescens Varieties Under Salt Stress

**Figure 1:**
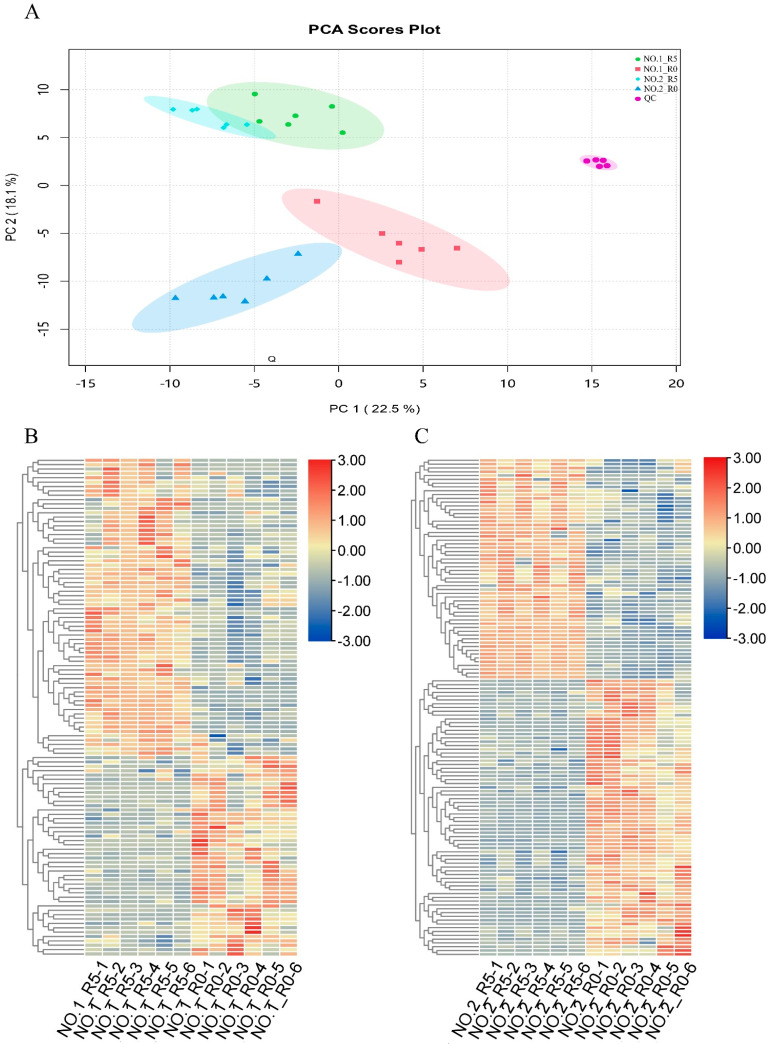
Global analysis of metabolomic data for *C. rigescens* under salt stress. (**A**) A principal component analysis (PCA) plot showing the changes in the metabolome in response to salt stress in Lvping NO.1 and Lvping NO.2. R5 and R0 means roots after 5 days salt treatment or without, and the number behind them means replicates. The same below. (**B**,**C**) Cluster analysis of all differentially abundant metabolites (DAMs) in Lvping NO.1 (**B**) and Lvping NO.2 (**C**). Red and blue colors indicate up- and down-regulated metabolites, respectively. The fold change (FC) in metabolite abundance under salt stress was used to generate clusters. The clusters were generated using TBtools software (v2.119) after Z-score scaling.

To determine the differences in metabolite compounds between salt-sensitive and salt-tolerant varieties of *C. rigescens* under salt stress, we performed metabolic profiling analysis of seedlings grown under control and NaCl treatment using an untargeted metabolomics method. The PCA scores plot showed that PC1 (22.5%) and PC2 (18.1%) separated the two varieties and the control and treatment groups (Figure 1A), indicating variation among the samples. Overview heatmaps showed differences in DAMs between the two varieties (Figure 1B,C). A total of 114 DAMs (46 down-regulated and 68 up-regulated under salt stress relative to the control) were detected in Lvping NO.1, and 131 DAMs (73 down-regulated and 58 up-regulated) were detected in Lvping NO.2 (Table S1). These results showed that salt stress induces changes in metabolite accumulation and that some DAMs differ between the two varieties. 

### 3.2. Analysis of the Overlap in DAMs Between Two C. rigescens Varieties

**Figure 2:**
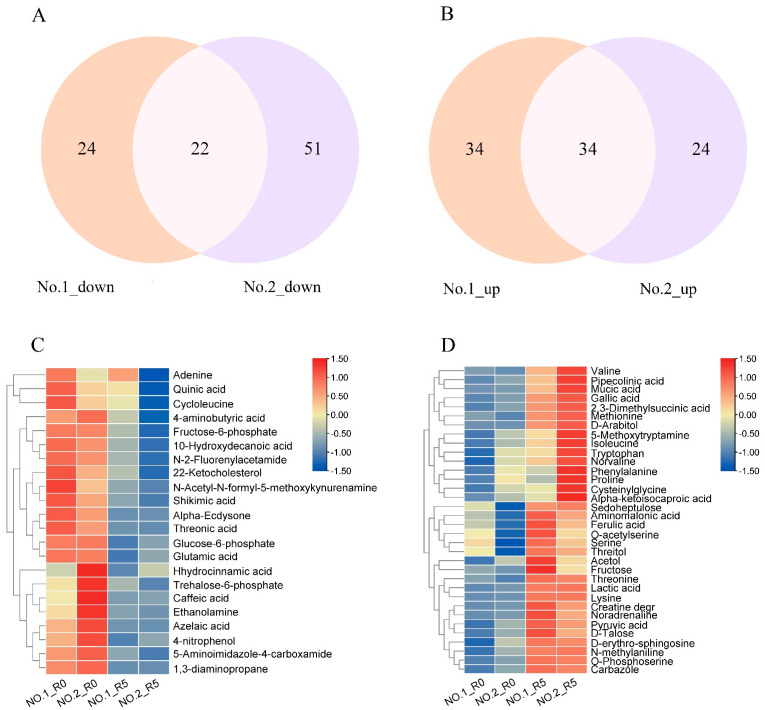
Analysis of the overlapping metabolites between two *C. rigescens* varieties. (**A**,**B**) Venn diagrams showing the overlap in down-regulated (**A**) and up-regulated (**B**) metabolites in *C. rigescens* variety Lvping NO.1 and Lvping NO.2 under NaCl treatment. (**C**,**D**) Heatmap of overlapping down-regulated (**C**) and up-regulated (**D**) metabolites in *C. rigescens* varieties NO.1 and NO.2 under NaCl treatment. Each heatmap was generated using TBtools software (v2.119) after Z-score scaling and log_2_ transformed changed ratio analysis. Red and blue colors indicate up- and down-regulated metabolites, respectively.

To further explore the differences and overlap between the DAMs in the two varieties, we generated a Venn diagram and heatmap (Figure 2). Twenty-two metabolites were down-regulated, and 34 metabolites were up-regulated in response to salt stress in both varieties of *C. rigescens* (Figure 2A,B). These included six amino acids (valine, phenylalanine, isoleucine, tryptophan, threonine, and serine), and metabolites involved in phenylalanine metabolism and phenylpropanoid biosynthesis like pyruvic acid and ferulic acid (Figure 2C), hydrocinnamic acid, shikimic acid, and quinic acid (Figure 2D). More DAMs involved in amino acid biosynthesis, phenylalanine metabolism, and phenylpropanoid biosynthesis were up-regulated than down-regulated.

### 3.3. KEGG Pathway Enrichment Analysis of DAMS in the Two C. rigescens Varieties

**Figure 3:**
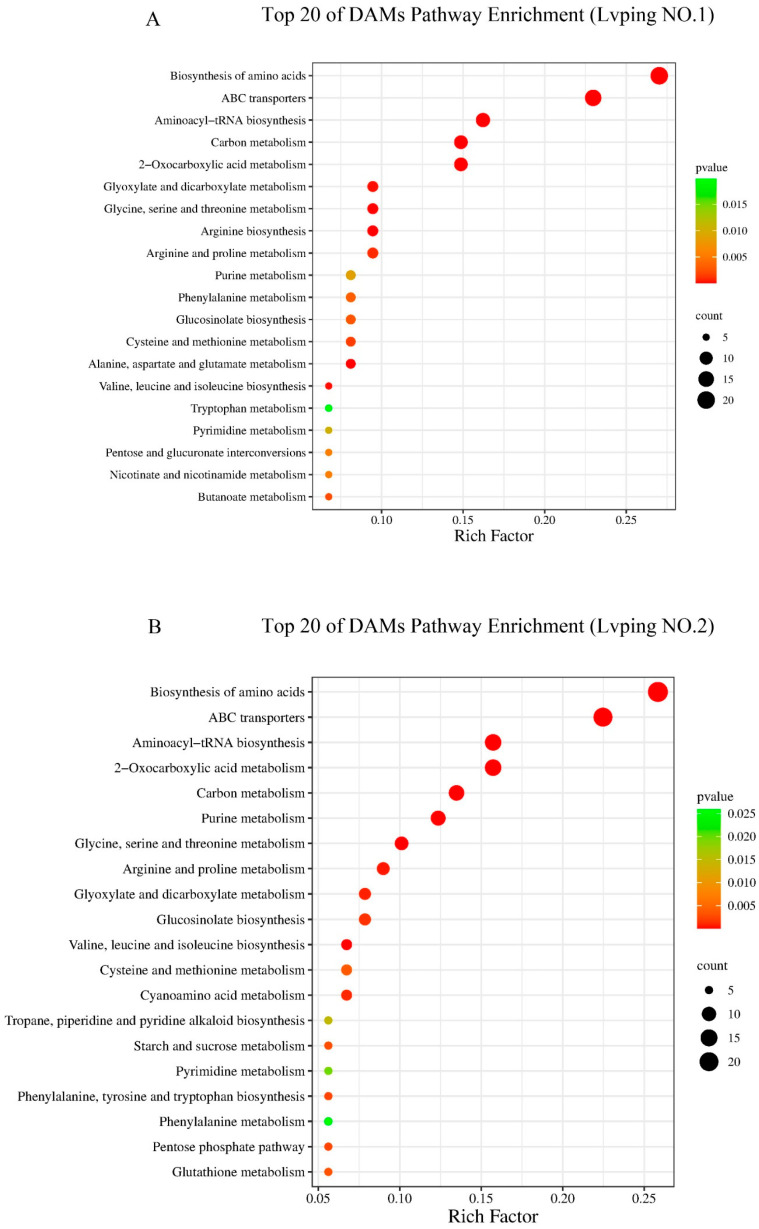
KEGG pathway enrichment analysis of the top 20 DAMs. (**A**,**B**) KEGG analysis of DAMs in Lvping NO.1 (**A**) and Lvping NO.2 (**B**). Count indicates how many DAMs each pathway contains. Rich factor represents the ratio of DAMs within the pathway to the total number of annotated metabolites in the pathway. The intensity of the dot color represents the *p* value; red indicates a smaller *p* value and green indicates a larger *p* value.

To further explore the functions of the DAMs under salt stress, we performed KEGG pathway enrichment analysis of DAMs in the two varieties; the top 20 enriched pathways are shown in Figure 3A,B. The top five KEGG pathways were the same in both varieties: biosynthesis of amino acids (ko01230), ABC transporters (ko02010), aminoacyl-tRNA biosynthesis (ko00970), carbon metabolism (ko01200), and 2-oxocarboxylic acid metabolism (ko01210). Of the top 10 pathways, arginine biosynthesis (ko00220) and glucosinolate biosynthesis (ko00966) were unique to Lvping NO.1 and Lvping NO.2. Of the remaining 10 pathways, five were unique to Lvping NO.1, namely butanoate metabolism (ko00650), nicotinate and nicotinamide metabolism (ko00760), pentose and glucuronate interconversions (ko00040), tryptophan metabolism (ko00380), and alanine, aspartate, and glutamate metabolism (ko00250). The following six pathways were unique to Lvping NO.2: cyanoamino acid metabolism (ko00460), glutathione metabolism (ko00480), pentose phosphate pathway (ko00030), starch and sucrose metabolism (ko00500), phenylalanine, tyrosine, and tryptophan biosynthesis (ko00400), and tropane, piperidine, and pyridine alkaloid biosynthesis (ko00960). In conclusion, the top 10 pathways were the same in the two varieties except arginine biosynthesis (ko00220) and glucosinolate biosynthesis (ko00966), indicating that the differences in the salt tolerance mechanism between the two varieties are small.

### 3.4. Profiling of Metabolites and the Corresponding DEGs Involved in Amino Acid Biosynthesis

**Figure 4:**
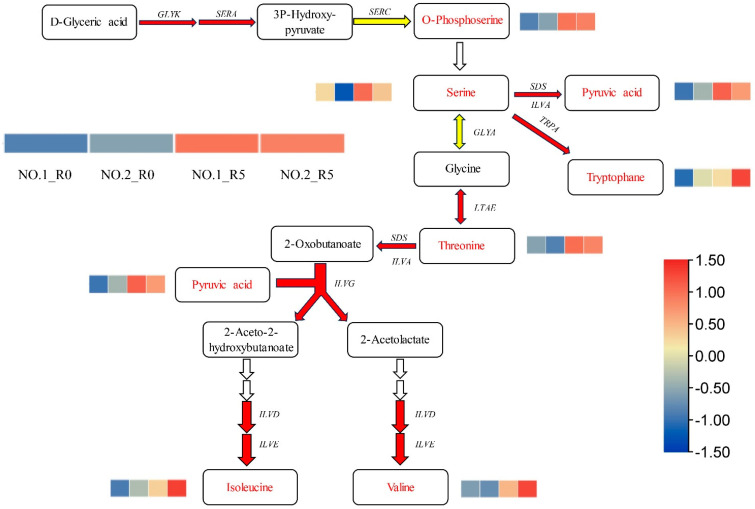
Metabolites and genes involved in amino acid biosynthesis in two *C. rigescens* varieties under salt treatment. Red arrows indicate up-regulated genes and red font indicates up-regulated metabolites. Yellow arrows indicate that some genes are up-regulated and some are down-regulated. The figure was generated using the FC values for each DAM and differentially expressed gene (DEG). The order of each heatmap from left to right is Lvping NO.1_R0, Lvping NO.2_R0, Lvping NO.1_R5, and Lvping NO.2_R5. The darker the color, the bigger the FC.

To further explore key amino acids regulating salt tolerance in *C. rigescens*, we investigated changes in the accumulation of amino acids and metabolites (Figure 4 and Table S1). We found that several metabolites related to amino acid biosynthesis, namely, o-phosphoserine, serine, pyruvic acid, tryptophane, threonine, isoleucine, and valine, accumulated in both varieties after salt stress. The contents of ornithine (Log2 FC = 1.98) and glycine (Log2 FC = 1.43) were higher in Lvping NO.2 after salt treatment but remained the same relative to the control in Lvping NO.1 (Table S1). D-Glyceric acid (Log2 FC = 0.62) was down-regulated in Lvping NO.2, but its content did not differ significantly compared with the control in Lvping NO.1 (Table S1). These results indicate that amino acids may play an indispensable role in salt tolerance in *C. rigescens.*

**Figure 5:**
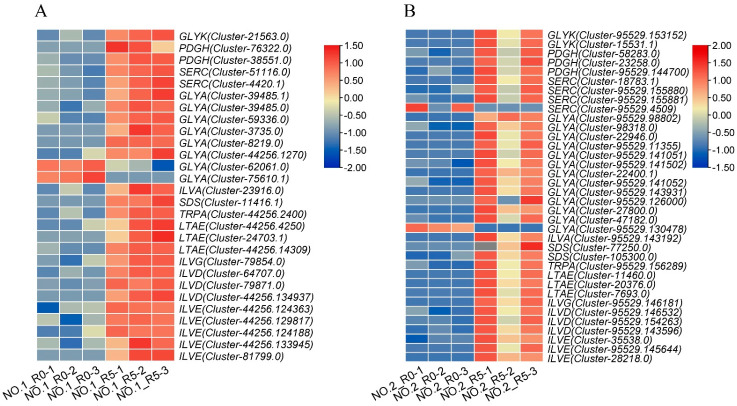
Heatmap of DEGs involved in amino acid biosynthesis pathways. (**A**,**B**) Heatmaps of DEGs involved in amino acid biosynthesis pathways in Lvping NO.1 (**A**) and Lvping NO.2 (**B**). The figure was generated using the FC values for each DEG. The clusters were generated using TBtools software (v2.119) after Z-score and log2 transformed changed ratio analysis. *GLYK: D-glycerate 3-kinase; PGDH: D-3-phosphoglycerate dehydrogenase; SERC: Phosphoserine aminotransferase; GLYA: Glycine hydroxymethyltransferase; ILVA: Threonine dehydratase; SDS: L-serine/L-threonine ammonia-lyase; TRPA: tryptophan synthase beta chain; LTAE: Threonine aldolase; ILVG: Acetolactate synthase I/II/III large subunit; ILVD: Dihydroxy-acid dehydratase; ILVE: Branched-chain amino acid aminotransferase*.

In order to determine the transcriptome gene expression and regulation of the DAMs mentioned above, we performed transcriptome analysis of the roots of the two varieties in parallel with the metabolome assay. The expression of genes involved in the amino acid biosynthesis pathway, except *phosphoserine aminotransferase* (*SERC*), was similar in the two varieties (Figure 5A,B). Twenty-six genes were up-regulated and two were down-regulated in Lvping NO.1, whereas thirty-four genes were up-regulated and two were down-regulated in Lvping NO.2 (Figure 5A,B). All *SERC* genes were up-regulated in Lvping NO.1, but one of these genes was down-regulated in Lvping NO.2. Moreover, some *glycine hydroxymethyltransferase* (*GLYA*) genes were up-regulated and some were down-regulated in the two varieties compared with the control (Figure 5A,B). The up-regulated genes in both varieties may be associated with DAMs involved in amino acid biosynthesis and may have potential in exploring the *C. rigescens* salt tolerance mechanism. 

### 3.5. Profiling of Metabolites and Differentially Expressed Genes Involved in Phenylalanine Biosynthesis and Metabolism and Phenylpropanoid Biosynthesis

**Figure 6:**
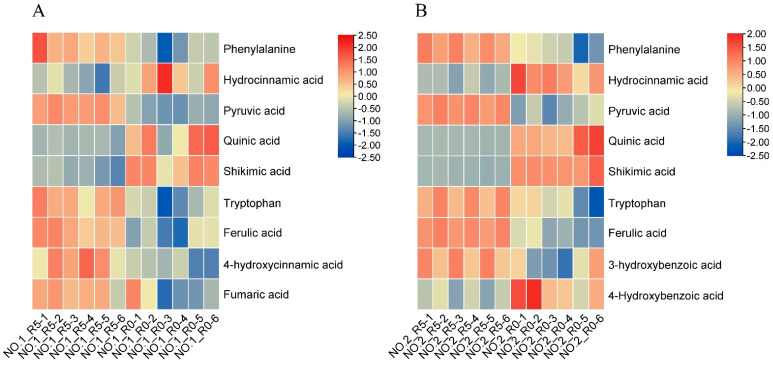
Heatmap of DAMs assigned to the pathway biosynthesis and metabolism of phenylalanine and to phenylpropanoid biosynthesis. (**A**,**B**) Heatmap of DAMs assigned to the pathways biosynthesis and metabolism of phenylalanine and phenylpropanoid biosynthesis in Lvping NO.1 (**A**) and Lvping NO.2 (**B**). The heatmaps were generated using TBtools software (v2.119) after Z-score and log_2_ transformed changed ratio analysis.

Eleven DAMs were assigned to biosynthesis and metabolism of phenylalanine and phenylpropanoid biosynthesis. Seven DAMs (phenylalanine, hydrocinnamic acid, pyruvic acid, quinic acid, shikimic acid, tryptophan, and ferulic acid) were common to both varieties, two metabolites (4-hydroxycinnamic acid and fumaric acid) were specific to Lvping NO.1 (Figure 6A), and two metabolites (3-hydroxybenzoic acid and 4-hydroxybenzoic acid) were specific to Lvping NO.2 (Figure 6B). More than half of the DAMs increased in abundance in both varieties after salt stress (six of nine DAMs in Lvping NO.1 and five of nine DAMs in Lvping NO.2).

A total of 147 DEGs involved in phenylalanine biosynthesis and metabolism and phenylpropanoid biosynthesis were explored, namely, 86 DEGs in Lvping NO.1 and 61 DEGs in Lvping NO.2, and most of them were up-regulated (52 of 86 genes in NO.1 and 47 of 61 in NO.2) compared with the control (Table 1). The expression pattern of DEGs was different in each variety. *PAL*, the gateway enzyme of the general phenylpropanoid pathway, was up-regulated only in Lvping NO.1 (Table 1). *Caffeoyl-CoA O-methyltransferase* (*CCoAOMT*), *aspartate-prephenate aminotransferase*, *anthranilate synthase component I*, *COMT*, and *shikimate O-hydroxycinnamoyltransferase* (*HCT*) were also only up-regulated in Lvping NO.1 (Table 1). *Histidinol-phosphate aminotransferase*, *tyrosine aminotransferase* (*TAT*), and *3-phosphoshikimate 1-carboxyvinyltransferase* were only up-regulated in Lvping NO.2 (Table 1). *Anthranilate phosphoribosyltransferase* genes were down-regulated in Lvping NO.1, and some were down-regulated and some were up-regulated in Lvping NO.2 (Table 1). *Cinnamyl-alcohol dehydrogenase* genes and *β-glucosidase* genes were up-regulated, and others were down-regulated in Lvping NO.1 but were only up-regulated in Lvping NO.2. Some *chorismate synthase* (*AOC3*), *3-hydroxybutyryl-CoA dehydrogenase* (*PAAH*), and *peroxidase* genes were up-regulated and others were down-regulated in both varieties. These differences in gene expression may explain why Lvping NO.2 has a higher salt tolerance than Lvping NO.1.

### 3.6. Validation of RNA-Seq Results with qRT-PCR

**Figure 7:**
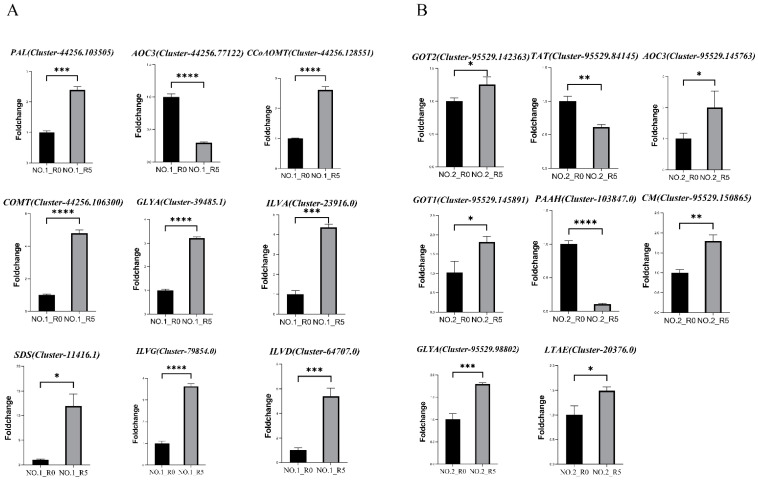
Analysis of candidate gene expression using qRT-PCR. (**A**,**B**) Nine candidate genes were chosen from Lvping NO.1 (**A**) and Lvping NO.2 (**B**) and analyzed by qRT-PCR to validate the expression patterns from the transcriptome data. Gene abbreviations are consistent with those in Figure 5 and Table 1. *, *p* < 0.05; **, *p* < 0.01; ***, *p* < 0.001; ****, *p* < 0.0001. Fold change means data calculated after 2^−ΔΔCt^.

To validate the gene expression patterns from RNA-seq, nine and eight genes were randomly selected in Lvping NO.1 and Lvping NO.2 from both treated and control samples for qRT-PCR analysis (Figure 7). *AOC3* (FC: 0.29) in Lvping NO.1 and *TAT* (FC: 0.62) and *PAAH* (FC: 0.10) in Lvping NO.2 were down-regulated. The remaining genes, *PAL* (FC: 2.39), *CCoAOMT* (FC: 2.62), *COMT* (FC: 4.80), *GLYA* (FC: 3.21), *ILVA* (FC: 4.36), *SDS* (11.96), *ILVG* (FC: 3.63), and *ILVD* (FC: 5.39) in Lvping NO.1, and *GOT2* (FC: 1.25), *AOC3* (FC: 2.00), *GOT1* (FC: 1.81), *chorismate mutase* (*CM*) (FC: 1.79), *GLYA* (Cluster-95529.98802) (FC:1.79), and *LTAE* (FC: 1.50) in Lvping NO.2 were up-regulated. These expression patterns align closely with those observed in the RNA-seq data (Figure 5, Table 1), affirming the reliability of the RNA-seq results.

## 4. Discussion

In China, there is widespread soil salinization, with salinized land accounting for about 4% of the global total [40]. The growth of plants is severely inhibited by saline environments; therefore, investigating plant salt tolerance mechanisms is imperative. Members of the genus *Carex*, one of the largest genera in Cyperaceae [41], are important native turfgrasses. *C. rigescens* possesses tolerance to multiple abiotic stresses [25]. A previous study comparing the salt stress responses and tolerances of Lvping NO.1 and Lvping NO.2 found that Lvping NO.2 has a higher tolerance to salt stress [26]. Here, we performed untargeted metabolomics combined with transcriptome analysis of these two *C. rigescens* varieties to investigate the mechanisms underlying the differences in salt stress tolerance. We identified a large number of DAMs, especially metabolites related to amino acid biosynthesis, phenylalanine biosynthesis, and metabolism and phenylpropanoid biosynthesis, which also corresponded to changes in the expression of DEGs identified by analysis of transcriptome data. Finally, we explored potential metabolites and genes related to salt tolerance, which may contribute to further elucidate the salt tolerance mechanisms of *C. rigescens*.

Amino acids have attracted attention due to their roles in plant growth and stress tolerance [42]. For example, the accumulation of amino acids has been found to enhance the drought tolerance of wheat and rice [43,44]. In this study, we found that six amino acids were up-regulated in two *C. rigescens* varieties after NaCl treatment, which was consistent with oat (*Avena sativa* L.) under salt stress [45]. In addition, the accumulation of serine was previously found to enhance the content of the important osmotic adjustment metabolite, betaine, in plants, which mitigates the damage caused by abiotic stress [46]. In this study, we found that both serine and its indispensable biosynthesis gene *D-3-phosphoglycerate dehydrogenase* (*PGDH*) [47] were up-regulated after NaCl stress in both varieties of *C. rigescens*, indicating that there may be a positive relationship between the accumulation of serine and salt tolerance in *C. rigescens*. Additionally, other research also found that the expression of *PGDH* plays an important role in sugar beet (*Beta vulgaris*) salt tolerance [48]. Furthermore, aromatic amino acids, including phenylalanine, tryptophan, and tyrosine, have been shown to directly and indirectly enhance the stress tolerance of plants [49]. In Lvping NO.1 and Lvping NO.2, the contents of phenylalanine and tryptophan increased after 5 days of salt treatment, and genes associated with phenylalanine biosynthesis or metabolism were also up-regulated, such as *PAL*, *3-hydroxybutyryl-CoA dehydrogenase*, and *CM*, which play crucial roles in phenylpropanoid metabolism or the accumulation of phenylpropionic acid-like products [50,51,52]. This observation suggests that *C. rigescens* might induce the biosynthesis of aromatic amino acids and secondary metabolites to adapt to salt stress conditions. Ornithine, one of the precursors of proline, has been found to be induced by various environmental stresses [53]. In this study, we found that ornithine was up-regulated in Lvping NO.2, but its expression was unchanged in Lvping NO.1 compared with the control. In addition, Lvping NO.2 had a higher proline abundance than Lvping NO.1 (Table S1). This may explain why Lvping NO.2 has a better salt tolerance than Lvping NO.1 [27]. To sum up, the changes in genes and metabolites involved in amino acid biosynthesis support the hypothesis that this pathway plays a vital role in *C. rigescens* salt tolerance.

Phenylpropanoid compounds have been shown to confer plants with better tolerance to multiple stresses such as drought, salinity, and heat [54]. According to the transcriptome analysis (Table 1), there were more DEGs (87 genes) in Lvping NO.1 than in Lvping NO.2 (61 genes). This finding was consistent with that of Li et al. [26], who proved that Lvping NO.1 has a higher phenylpropanoid biosynthesis. *HCT* genes, which are located at key branch points of the lignin and flavonoid pathways, play a role in plant responses to external stress [55]. However, *HCT* genes were down-regulated in Lvping NO.1, which was also consistent with wheat under salt stress [56], and were not differentially expressed in Lvping NO.2 compared with the control. This may indicate a negative correlation between the expression of *HCT* genes and salt tolerance of *C. rigescens*. *β-glucosidases*, as multi-functional enzymes, have been shown to enhance abiotic tolerance by enhancing the content of antioxidant flavonols in *Nicotiana benthamiana* and regulating the level of salicylic acid in rice [57,58,59]. In our study, we found that β-glucosidases were up-regulated in Lvping NO.2, and a large proportion (10 of 14 genes) were also up-regulated in Lvping NO.1. This may suggest a positive correlation between the expression of *β-glucosidases* and the salinity tolerance of *C. rigescens*. *Ferulate-5-hydroxylase* (*F5H*) is an important enzyme in syringyl lignin biosynthesis, catalyzing the synthesis of 5-hydroxyferulic acid from ferulic acid. Contrary to the increase in ferulic acid, foliar application of which was found to mitigate damage caused by salt stress, *F5H* expression decreased in response to salt stress in the two varieties, which was consistent with the decrease in *F5H* expression observed in wheat under salt stress [60,61]. This finding may indicate not only a negative correlation between *F5H* and salt tolerance, but also between the syringyl lignin content and salt tolerance in *C. rigescens*. As shown in Figure 6, most of the DAMs (7 of 9) were quite similar in the two varieties. However, the increase in the content of 4-hydroxycinnamic acid in Lvping NO.1 after salt treatment was consistent with the leaves of salt-sensitive rice varieties under salt stress [62]. This may strongly support the differences between two varieties and may act as a breakthrough to explore the reason for high salt tolerance in Lvping NO.2.

## 5. Conclusions

In conclusion, this study performed combined GC-MS and high-throughput sequencing to analyze the metabolome and transcriptome profiles of two *C. rigescens* varieties after salt treatment. Three pathways were found to be induced by salt stress: amino acid biosynthesis, phenylalanine biosynthesis and metabolism, and phenylpropanoid biosynthesis. Some metabolites and genes in the three pathways have even been demonstrated to function in crop abiotic stress. We suggest that the accumulation of ferulic acid and amino acids, in particular serine and phenylalanine, may positively mitigate damage caused by salt stress. Our findings have the potential to reveal new pathways, metabolites, and genes that regulate and improve the salt tolerance of *C. rigescens*, as well as be potential candidates for developing salt-tolerant crop varieties.

## Figures and Tables

**Table 1:** Information about DEGs involved in phenylalanine metabolism and biosynthesis and phenylpropanoid biosynthesis in two *C. rigescens* varieties.

		Lvping NO.1		Lvping NO.2	
Gene_Name	Annotation	Gene_id	FC	Gene_id	FC
*PAL*	*Phenylalanine ammonia-lyase*	Cluster-44256.103505	2.40	-	-
*GOT1*	*Aspartate aminotransferase, cytoplasmic*	Cluster-44256.8151	5.06	Cluster-95529.145891	8.19
*GOT2*	*Aspartate aminotransferase, mitochondrial*	Cluster-44256.135586	4.58	Cluster-95529.143830	10.76
				Cluster-95529.143831	6.44
				Cluster-95529.142363	8.79
				Cluster-16859.0	8.16
				Cluster-95529.11116	5.12
*HISC*	*Histidinol-phosphate aminotransferase*	-	-	Cluster-55824.0	7.31
*TAT*	*Tyrosine aminotransferase*	-	-	Cluster-95529.84145	−6.37
*PAT*	*Aspartate-prephenate aminotransferase*	Cluster-44256.60806	3.52	-	-
*4CL*	*4-Coumarate-CoA ligase*	Cluster-44256.1115	5.41	Cluster-95529.154551	8.93
		Cluster-65152.1	4.85	Cluster-77190.1	7.43
		Cluster-4013.1	5.28	Cluster-95529.11377	8.69
		Cluster-44256.90528	3.04	Cluster-95529.144189	6.60
				Cluster-95529.115301	6.49
				Cluster-95529.144191	7.65
*AOC3*	*Primary-amine oxidase*	Cluster-17998.0	6.08	Cluster-46891.0	7.71
		Cluster-44256.77122	−4.26	Cluster-95529.145763	8.63
				Cluster-95529.98458	−8.99
*HPD*	*4-hydroxyphenylpyruvate dioxygenase*	Cluster-44256.1169	6.79	Cluster-23538.2	7.42
		Cluster-44256.135595	8.65	Cluster-23538.1	8.03
		Cluster-44256.134939	3.94		
		Cluster-78695.0	−6.58		
*CCoAOMT*	*Caffeoyl-CoA* *O-methyltransferase*	Cluster-44256.128551	7.17	-	-
		Cluster-44256.142190	3.83	-	-
*AMIE*	*Amidase*	Cluster-44256.7300	3.65	Cluster-103487.0	6.85
*PAAH*	*3-hydroxybutyryl-CoA dehydrogenase*	Cluster-44256.281	4.57	Cluster-19576.0	7.39
		Cluster-29667.0	−6.95	Cluster-95529.17745	9.17
				Cluster-103847.0	−6.33
*AROF*	*3-deoxy-7-phosphoheptulonate synthase*	Cluster-44256.140245	5.73	Cluster-95529.18665	7.45
		Cluster-42655.0	4.54		
*TRPB*	*Tryptophan synthase beta chain*	Cluster-44256.2400	5.31	Cluster-95529.156289	7.28
*AROA*	*3-phosphoshikimate* *1-carboxyvinyltransferase*	-	-	Cluster-95529.155594	7.29
*AROC*	*Chorismate synthase*	Cluster-2755.0	7.23	Cluster-28537.1	7.57
*TRPD*	*Anthranilate phosphoribosyltransferase*	Cluster-75524.1	−8.71	Cluster-13667.1	8.10
		Cluster-95135.0	−3.28	Cluster-13667.0	9.00
				Cluster-95529.28021	−8.27
*TRPG*	*Anthranilate* *synthase component II*	Cluster-44256.140550	4.41	Cluster-95529.140478	7.91
*TRPE*	*Anthranilate synthase component I*	Cluster-20668.0	6.51	-	-
		Cluster-44256.104437	−6.68	-	-
*CM*	*Chorismate mutase*	Cluster-44256.138504	4.78	Cluster-95529.150866	6.39
		Cluster-44256.2200	4.11	Cluster-95529.150865	10.32
*COMT*	*Caffeic acid-O-methyltransferase*	Cluster-44256.106300	2.49	-	-
		Cluster-44256.52838	−2.89	-	-
*F5H*	*Ferulate-5-hydroxylase*	Cluster-44256.105355	−2.67	Cluster-95529.25846	−4.47
*HCT*	*Shikimate hydroxycinnamoyltransferase*	Cluster-44256.54073	−4.32	-	-
		Cluster-44256.68688	−4.62	-	-
*REF1*	*Coniferyl-aldehyde dehydrogenase*	Cluster-44256.129889	−5.95	Cluster-11814.0	9.06
		Cluster-38909.0	4.40		
*CAD*	*Cinnamyl-alcohol dehydrogenase*	Cluster-11624.1	8.32	Cluster-95529.4701	7.25
		Cluster-44502.0	4.16	Cluster-95529.4699	7.61
		Cluster-44256.16008	4.82	Cluster-95529.19277	8.36
		Cluster-44256.126247	7.01	Cluster-29171.0	8.76
		Cluster-44256.141092	5.10		
		Cluster-44256.1876	7.43		
		Cluster-44256.29315	1.90		
*BG*	*β-Glucosidase*	Cluster-53098.0	−7.24	Cluster-95529.140120	9.59
		Cluster-44256.95249	2.49	Cluster-11117.0	8.01
		Cluster-44256.106759	3.24	Cluster-95529.156418	9.19
		Cluster-44256.106754	2.67	Cluster-6928.0	8.38
		Cluster-44256.13564	7.11	Cluster-93594.0	8.79
		Cluster-44256.56968	3.50	Cluster-14033.0	8.00
		Cluster-44256.133126	5.27		
		Cluster-44256.1863	5.05		
		Cluster-44256.110055	7.34		
		Cluster-44256.82742	3.33		
		Cluster-44256.106624	7.50		
		Cluster-44256.104190	−3.78		
		Cluster-44256.76573	−3.05		
		Cluster-44256.95609	−2.78		
*PRDX6*	*Peroxiredoxin 6*	Cluster-44256.95610	−3.22	Cluster-95529.140059	7.51
		Cluster-44256.133837	4.87	Cluster-95529.140058	10.12
				Cluster-13772.0	6.99
				Cluster-120365.0	5.77
*PX*	*Peroxidase*	Cluster-79112.0	−6.72	Cluster-95529.127641	5.02
		Cluster-44256.16716	3.54	Cluster-95529.136496	5.51
		Cluster-44256.110469	3.08	Cluster-95529.107100	5.09
		Cluster-44256.95843	6.57	Cluster-95529.125510	−5.89
		Cluster-44256.5261	6.61	Cluster-95529.125998	−4.96
		Cluster-44256.5263	7.30	Cluster-95529.29513	−8.41
		Cluster-44256.107519	5.27	Cluster-95529.108973	−9.77
		Cluster-44256.779	5.27	Cluster-95529.108277	−5.02
		Cluster-44256.109740	4.99	Cluster-95529.121703	−5.63
		Cluster-44256.31832	−2.59	Cluster-95529.108837	−5.58
		Cluster-44256.106374	−4.05	Cluster-95529.113607	−5.14
		Cluster-44256.106148	−3.96	Cluster-95529.112865	−5.16
		Cluster-44256.127711	−3.15		
		Cluster-44256.31662	−7.95		
		Cluster-44256.54631	−6.54		
		Cluster-44256.108206	−4.73		
		Cluster-44256.54052	−4.76		
		Cluster-44256.53240	−4.12		
		Cluster-44256.109273	−4.83		
		Cluster-44256.52883	−3.69		
		Cluster-44256.53650	−5.40		
		Cluster-44256.31658	−8.28		
		Cluster-44256.112274	−3.93		
		Cluster-44256.50645	−2.49		
		Cluster-44256.105887	−4.40		
		Cluster-44256.108233	−6.24		

“FC” means log2(fold change). “-” means that a gene was not detected in the variety.

## Data Availability

The data in this study will be available upon request to the corresponding author.

## Supplementary Materials

The following supporting information can be downloaded at: https://www.mdpi.com/article/10.3390/plants13212984/s1, Table S1: DAMs in Lvping NO.1 and Lvping NO.2 ranked by fold change difference in abundance under salt stress; Table S2: The software and thresholds used for functional annotation of unigenes; Table S3: Gene-specific primers used for qRT-PCR validation of RNA-seq results.
