# Supplementary material for: Comparative Metabolome and Transcriptome Analyses Reveal Molecular Mechanisms Involved in the Responses of Two Carex rigescens Varieties to Salt Stress
Source: Plants (Basel). 2024 Oct 25;13(21):2984. doi: 10.3390/plants13212984 (PMC11548242; doi:10.3390/plants13212984)
Supplement: Supplementary file 1 [file plants-13-02984-s001.zip › plants-3194334-supplementary/Table S2.pdf]

Database	Brief introduction	Software	Parameters
Nr	NCBI non-redundant protein sequences	diamond v0.8.22	e-value = $1e^{-5}$, --more-sensitive
Nt	NCBI nucleotide sequences	NCBI blast 2.2.28+	e-value = $1e^{-5}$
Pfam	Protein family.<br>Website: http://pfam.sanger.ac.uk/	HMMER 3.0 package, hmmscan	e-value = 0.01
KOG/COG	COG: Clusters of Orthologous Groups of proteins<br>KOG: euKaryotic Ortholog Groups<br>Website: http://www.ncbi.nlm.nih.gov/COG/	diamond v0.8.22	e-value = $1e^{-3}$, --more-sensitive
Swiss-Prot	A manually annotated and reviewed protein sequence database<br>Website: http://www.ebi.ac.uk/uniprot/	diamond v0.8.22	e-value = $1e^{-5}$, --more-sensitive
KEGG	Kyoto Encyclopedia of Genes and Genomes<br>Website: http://www.genome.jp/kegg/	KAAS, KEGG Automatic Annotation Server	e-value = $1e^{-10}$
